# Immune-Related Four-lncRNA Signature for Patients with Cervical Cancer

**DOI:** 10.1155/2020/3641231

**Published:** 2020-11-12

**Authors:** Jianfeng Zheng, Benben Cao, Xia Zhang, Zheng Niu, Jinyi Tong

**Affiliations:** ^1^Department of Obstetrics and Gynecology, Affiliated Hangzhou Hospital, Nanjing Medical University, Hangzhou, Zhejiang Province 310008, China; ^2^Department of Obstetrics and Gynecology, Hangzhou Women's Hospital, Hangzhou, Zhejiang Province 310008, China; ^3^Department of fourth Clinical Medical College, Zhejiang Chinese Medical University, Hangzhou, Zhejiang Province 310006, China; ^4^Department of Obstetrics and Gynecology, Affiliated Hangzhou First People's Hospital, Zhejiang University of Medicine, Hangzhou, Zhejiang Province 310006, China

## Abstract

Cervical cancer (CC) is a common gynecological malignancy for which prognostic and therapeutic biomarkers are urgently needed. The signature based on immune-related lncRNAs (IRLs) of CC has never been reported. This study is aimed at establishing an IRL signature for patients with CC. A cohort of 326 CC and 21 normal tissue samples with corresponding clinical information was included in this study. Twenty-eight IRLs were collected according to the Pearson correlation analysis between the immune score and lncRNA expression (*p* < 0.01). Four IRLs (BZRAP1-AS1, EMX2OS, ZNF667-AS1, and CTC-429P9.1) with the most significant prognostic values (*p* < 0.05) were identified which demonstrated an ability to stratify patients into the low-risk and high-risk groups by developing a risk score model. It was observed that patients in the low-risk group showed longer overall survival (OS) than those in the high-risk group in the training set, valid set, and total set. The area under the curve (AUC) of the receiver operating characteristic curve (ROC curve) for the four-IRL signature in predicting the one-, two-, and three-year survival rates was larger than 0.65. In addition, the low-risk and high-risk groups displayed different immune statuses in GSEA. These IRLs were also significantly correlated with immune cell infiltration. Our results showed that the IRL signature had a prognostic value for CC. Meanwhile, the specific mechanisms of the four IRLs in the development of CC were ascertained preliminarily.

## 1. Introduction

Cervical cancer (CC) is a malignant gynecologic tumor threatening the health of women. The morbidity and mortality for CC rank fourth worldwide among women [1]. Infection with high-risk human papillomavirus (HPV), especially HPV16 and HPV18, is the main etiologic risk factor for CC and plays an important role in diagnostic tests [2]. Surgery is the main treatment method for CC in early stages while advanced-stage CC can be treated with radiotherapy, chemotherapy, or concurrent chemoradiation, thereby improving the survival rate of CC patients [3]. However, a considerable number of CC patients have poor prognosis due to metastasis or recurrence within two years after treatment [4]. Hence, effective prevention to reduce morbidity and individual treatments to improve the prognosis of CC are important for obstetricians and gynecologists.

The immune system can recognize tumor antigens expressed on the surface of tumor cells. It generates an immune response via the activation of effector cells and triggers the release of a series of effector molecules to attack and eliminate tumor cells and to inhibit tumor growth [5]. The immune imbalance in the tumor microenvironment plays an important role in the occurrence and development of cancer. With the development of cellular molecular biology and immunology, immunotherapy has become a new treatment approach for cervical cancer [6]. Tumor immunotherapy acts mainly by increasing the immunogenicity of tumor cells and the effect of cell damage sensitivity and stimulates and enhances the antitumor immune response, with the aid of biological agents; doping effects of immune cells and molecules into the body, together, the body's immune system can not only kill cancer cells remaining small but also can prevent tumor metastasis and recurrence [7].

Long noncoding RNAs (lncRNAs), defined arbitrarily as transcripts lacking protein-coding potential, are RNAs with more than 200 nucleotides [8]. lncRNAs are not only associated with the invasion, migration, and proliferation of CC but also are involved in autophagy and epithelial-mesenchymal transition (EMT) [9]. Emerging studies have demonstrated that lncRNAs also modulate the immune response to tumors. For instance, the downregulated lncRNA AGER has been demonstrated to be closely related to T cell status in lung cancer [10]. lncRNA GM16343 is regulated by interleukin 36*β* to strengthen the antitumor immune response of CD8^+^ T cells [11]. lncRNA LINK-A has been demonstrated to downregulate antigen presentation and intrinsic suppression in triple-negative breast cancer [12]. lncRNAs can also affect the development of cancer by regulating NK cells. lncRNA GAS5 can inhibit tumor growth, and the overexpression of GAS5 can regulate miR-544/RUNX3 to enhance the killing effect of NK cells in liver cancer [13]. lncRNAs can also modulate tumor immunity by regulating Treg cells. The expression of miR-448 can be increased by interfering with lncRNA SNHG1, which downregulates the expression of indoleamine 2,3-dioxygenase (IDO), inhibits the differentiation of Treg cells, and reduces the immune escape of breast cancer cells [14]. EGFR is an important member of the tyrosine kinase receptor family. By binding to EGFR specifically, LNC-EGFR promotes Treg cell differentiation and promotes the immune escape of liver cancer cells [15]. lncRNAs can affect the tumor microenvironment and thus play an important role in immunotherapy. Nonetheless, the effect research on immune-related lncRNAs in CC is rarely reported.

The purpose of our research was to identify an IRL signature, which might serve as prognostic and therapeutic biomarkers in CC. We developed a prognostic signature and mechanisms of IRLs using single-sample gene set enrichment analysis (ssGSEA), survival analysis, a Cox regression risk model, gene set enrichment analysis (GSEA), ceRNA network, and other analysis methods.

## 2. Materials and Methods

### 2.1. Datasets and Preprocessing

The RNA sequencing profiles associated with CC were obtained from The Cancer Genome Atlas (TCGA) [16] (https://toil.xenahubs.net), which consisted of 306 CC and 13 normal tissue samples. The low-expression genes were filtered, and the genes whose expression level was greater than 0 in more than a third of the samples were retained. Additionally, the RNAs were identified as mRNAs or lncRNAs based on their annotation information in the GENCODE database [17] (https://www.gencodegenes.org/). GPL570 (HG-U133_Plus_2) Affymetrix Human Genome U133 Plus 2.0 Array platform was used to obtain the microarray dataset GSE6791 from the Gene Expression Omnibus (GEO) repository (Gene Expression Omnibus (GEO), http://www.ncbi.nlm.nih.gov/geo/) [18], of which twenty CC and eight normal tissue samples were included. All 326 CC samples and 21 normal tissue samples were contained with corresponding clinical information. For the RNA sequencing profiles obtained from TCGA TARGET GTEx, empirical Bayes and linear regression along with Benjamini and Hochberg multiple comparison methods from the limma package [19] (version 3.10.3, http://www.bioconductor.org/packages/2.9/bioc/html/limma.html) were performed to gain adjusted *p* value and |logFC| (adj.*p*.value < 0.05, ∣logFC | >0.585). For GSE6791, GEO2R (http://www.ncbi.nlm.nih.gov/geo/geo2r/) was used with the SeqMap [20] tool to map probes to mRNA and lncRNA sequences. The immune-related genes (IRGs) were downloaded from the InnateDB database (http://www.innatedb.com) [21]. Foc`using on screening genes that were up- and downregulated consistently, we used Venn analysis to select the intersection genes of the aforementioned datasets. Single-sample gene set enrichment analysis (ssGSEA) [22] was used to identify the immune scores (IS) of each sample. Pearson's correlation coefficient between lncRNAs and IS was calculated for each corresponding samples to identify immune-related lncRNAs (IRLs) (*p* < 0.01).

### 2.2. Signature Development of IRLs

Univariate Cox regression analysis with hazard ratio (HR) was gained from overall survival (OS) and OS time from the candidate IRLs. HR > 1 indicated that expression was higher and the risk and the survival rate were lower. lncRNAs that are upregulated in the tumor should, in principle, have an HR greater than 1. The Kaplan–Meier analysis was generated by survminer (version 0.4.3) in the R package based on the expression value, survival time, and survival status to determine the optimal cut point. The log-rank test was performed based on survival (version 2.42-6) in the R package to sort the IRLs with a significant prognostic value (*p* < 0.05); then, the survival curves were drawn. Multivariate Cox regression analysis was performed based on the expression value, OS, and OS time of IRLs in each sample. Subsequently, the individual prognostic risk model for corresponding samples was established. Expr_gene_ indicated the expression of corresponding IRL for each sample. All the samples were divided into the low-risk and high-risk groups according to the median of risk scores of the following study. All the samples in TCGA were regarded as a total set. Training and valid sets were constructed by dividing the total set equally into two parts to validate the risk score formula. The Kaplan–Meier survival curves of all three sets were drawn to determine the prognostic difference between the risk groups. The one-year, two-year, and three-year survival receiver operating characteristic (ROC) curves predicted by the risk model were drawn.

### 2.3. Clinical and Pathological Characteristics of the Risk Score Model

Clinical and pathological characteristics, including age, pathologic M, pathologic N, pathologic T, clinical stage, neoplasm histologic grade, neoplasm cancer status, primary therapy outcome success, radiation therapy, tobacco smoking history, along with the risk score, the immune score, and the expression of IRLs in each sample were included in the analysis for illustration of a heat map. We observed the differences in clinical pathological characteristics in risk groups or the risk score of clinical pathological characteristics using Student's *t*-test. A scatter plot was drawn using GraphPad Prism 5 [23] based on significant clinicopathological characteristics.

### 2.4. Nomogram Model Construction and Visualization

Univariate Cox regression analyses were performed, respectively, based on the risk groups and clinicopathological factors including age, cancer type (adenocarcinoma and squamous cell carcinoma), FIGO stage, TNM stages, histologic grade, radiation therapy, and smoking history. Multivariate Cox regression analysis was conducted on the factors with *p* < 0.05. Using the IRL signature along with the factors with *p* < 0.05, a nomogram was constructed to visualize the results of multivariate Cox regression analysis more clearly.

### 2.5. Gene Set Enrichment Analysis

To elucidate the biological differences between risk groups, a gene set enrichment analysis (GSEA) [24] of “c5.bp.v7.1.symbols.gmt” background was carried out using GSEA (version 4.0.3) software. A nominal *p* value < 0.05 (NOM *p* val < 0.05) was considered significant. We focused on selecting immune-related terms for display.

### 2.6. Evaluation of Infiltrating Immune Cells

To further observe the differences in the abundance of infiltrating immune cells of the risk groups, we used the CIBERSORT algorithm, a deconvolution method [25], coupled with LM22 that distinguished 22 immune cell subpopulations from CIBERSORT (a web server), and a heat map for all the samples was drawn using ggplot2 (version 3.2.1). Student's *t*-test was applied to find significant immune cell subpopulations in risk groups to chart violin plot by boxplot (version 0.3.2) in the R package.

### 2.7. Construction of an IRL-Associated ceRNA Network

The ceRNA network was constructed to explore the association among IRLs, miRNAs, and mRNAs based on the ceRNA hypothesis [26]. First, the Pearson correlation coefficient analysis between the IRLs and immune-related DEGs was performed to obtain lncRNA-mRNA pairs (*r* > 0 and adj.*p*.value < 0.01). The target miRNAs of IRLs were predicted using DIANA-LncBase v2 [27] (http://carolina.imis.athenainnovation.gr/diana_tools/web/index.php?r=lncbasev2%2Findex-experimental). The mRNAs targeted by miRNAs were predicted by integrating miRWalk2.0 [28] (version:3.4.0, http://chianti.ucsd.edu/cytoscape-3.4.0/), miRanda, RNA22, and TargetScan databases. Only miRNA-mRNA pairs recognized by all four databases were considered candidate targets. Subsequently, the ceRNA network based on the same miRNA of the lncRNA-miRNA and miRNA-mRNA pairs was established and visualized using Cytoscape (version:3.4.0, http://chianti.ucsd.edu/cytoscape-3.4.0/) [29]. Sorting the mRNA with a significant prognostic value (*p* < 0.05) in the ceRNA network, the Kaplan–Meier analysis was generated using survminer (version 0.4.3) to determine the optimal cut point, and the log-rank test was performed based on survival (version 2.42-6) in the R package.

## 3. Results

### 3.1. Differential Analysis of Genes

**Figure 1:**
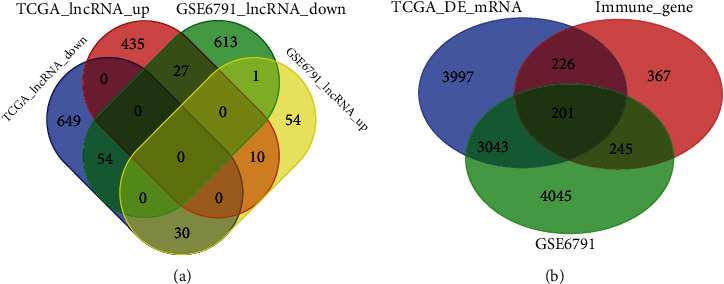
Venn analysis. (a) Venn diagram of differential lncRNAs. (b) Venn diagram of differential mRNA and immune-related genes.

In the aggregate, 1995 differentially expressed lncRNAs, with corresponding clinical information, were extracted from the TCGA and GSE6791 databases mentioned above. Of these, 567 lncRNAs were highly expressed and 1428 lncRNAs were expressed at low levels in the CC samples (Table 1). A total of 64 lncRNAs were obtained from the intersection of the two databases (Figure 1(a)), among which 10 lncRNAs were consistently upregulated and 54 lncRNAs were consistently downregulated in the two databases. A total of 1040 IRGs were identified from the InnateDB. The ssGSEA analysis was based on 201 IRGs obtained from the intersection of the TCGA and InnateDB databases (Figure 1(b)) to compute IS. Finally, a cohort of 28 IRLs was obtained based on the Pearson correlation coefficient analysis between the 64 lncRNAs and IS of 201 IRGs (*p* < 0.01).

### 3.2. Univariate Cox Regression and Kaplan–Meier Survival Analysis

**Figure 2:**
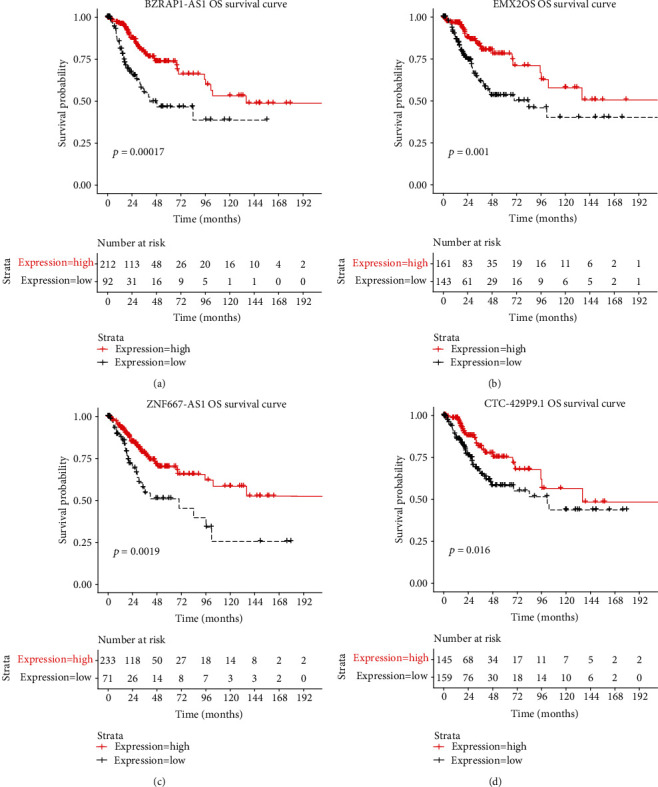
Kaplan–Meier curve of 4 IRLs.

Univariate Cox regression and Kaplan–Meier survival analysis were performed for the cohort of 28 IRLs. Four IRLs (BZRAP1-AS1, EMX2OS, ZNF667-AS1, and CTC-429P9.1) with low expression were found to be in accordance with our expectation and were then included in the signature development. The results are shown in Table 2. The Kaplan–Meier survival analysis revealed that four IRLs were related to OS in CC significantly (*p* < 0.05) (Figure 2). The four IRLs were defined as protective factors due to their HRvalue < 1, which showed that the high expression of the four IRLs was associated with lower OS.

### 3.3. Construction of the Prognostic Risk Model and Validation Using Four IRLs

A total of 304 TCGA samples were regarded as the total set so that there were both 152 samples in the training set and the valid set as described in the methods. The regression coefficient *β* was first generated from the training set (*β*_ZNF667−AS1_ = −0.152565, *β*_EMX2OS_ = −0.019887, *β*_BZRAP1−AS1_ = −0.17831, and *β*_CTC−429P9.1_ = 0.0096755; Table 3) to establish the risk score (RS) formula. Hence, the prognostic risk model for corresponding samples was established using the following formula:

(1) $$\begin{array}{c} \text{risk}\text{score}\left(\text{RS}\right)={\text{expr}}_{\text{BZRAP}1-\text{AS}1}*\left(-0.152565\right)+{\text{expr}}_{\text{EMX}2\text{OS}}*\left(-0.019887\right)+{\text{expr}}_{\text{BZRAP}1-\text{AS}1}*\left(-0.17831\right)+\text{expr}\text{CTC}-429\text{P}9.1*0.0096755. \end{array}$$

**Figure 3:**
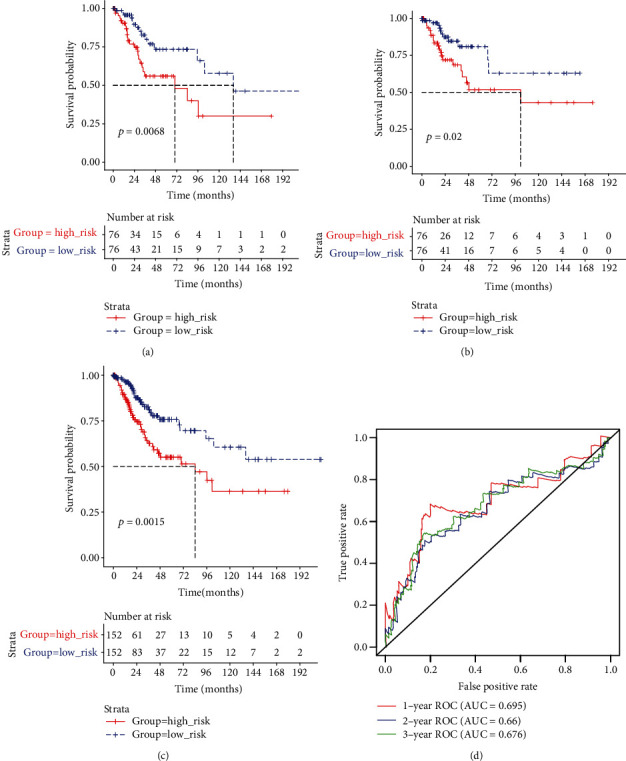
The 4-IRL prognostic risk model and validating. (a) Kaplan–Meier survival curves of OS among CC patients from different groups stratified by the signature in the training set, the valid set, and the total set. (b) Time-dependent receiver operating characteristic (ROC) curve for predicting overall survival (OS) of the risk model.

Expr_gene_ indicated the expression value of the corresponding IRL for each sample. An RS higher than the median was identified as a high-risk group while an RS lower than the medium was identified as a low-risk group. Using this approach, a risk model signature based on four IRLs was constructed, which was further validated in the total set and valid set using the same *β* to confirm the prediction potential of the 4-IRL signature. The Kaplan–Meier survival curves based on the log-rank test revealed that OS in the low-risk group was markedly longer than that in the other group in all three sets (*p*_Training−set_ = 0.0068, *p*_Valid−set_ = 0.02, and *p*_Total−set_ = 0.0015; Figures 3(a)–3(c)). The one-year, two-year, and three-year survival ROC curves predicted by the risk model indicated that the AUCs were larger than 0.65 (0.695, 0.66, and 0.676; Figure 3(d)), thus predicting that the risk score model could efficiently forecast over 65% of OS for CC patients. Therefore, the risk model signature based on four IRLs was accurate in predicting OS of CC patients.

### 3.4. Clinicopathological Characteristics of the Risk Score Model

**Figure 4:**
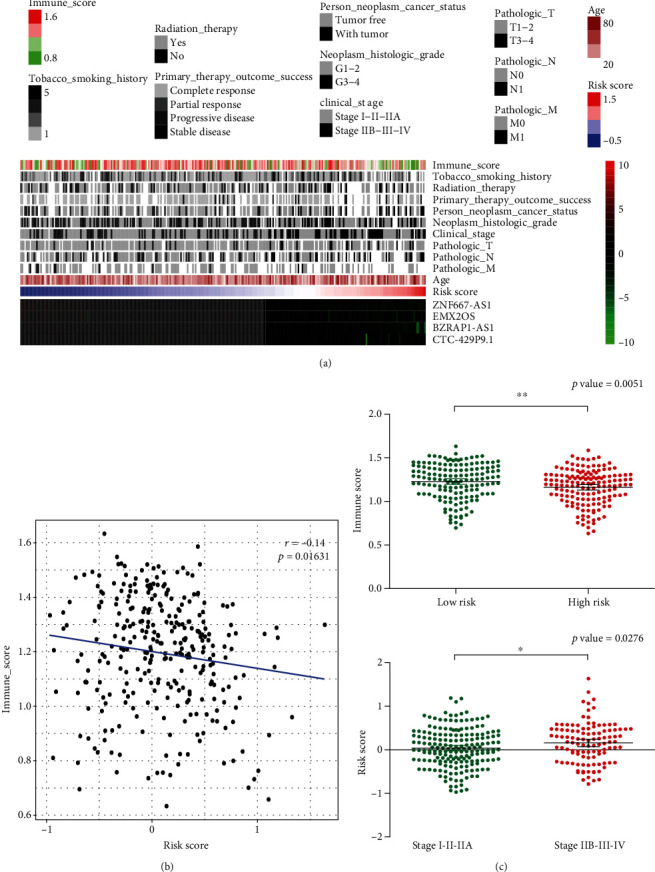
Clinical pathological characteristics of the risk score model. (a) Distribution of the immune score, clinicopathologic features, 4-IRL expression, and risk score. (b) Correlations between the risk score and immune score. (c) Immune score in different risk groups and risk score in tumor different stages.

A heat map was constructed by combining the expression values of four IRLs and their clinicopathological characteristics (Figure 4(a)). The higher the RS, the lower the IS. RS and IS values showed a significant negative correlation based on the scatterplot of the correlation coefficient (*r* = −0.14, *p* = 0.01631; Figure 4(b)). The scatterplot for the distribution of IS in risk groups showed that the IS of the high-risk group was significantly lower than that of the low-risk group (Figure 4(c)). Furthermore, the high expression of the four IRLs could be seen with low RS, which suggested that the upregulation of the four IRLs were associated with better prognosis. In contrast, the low expression of the four IRLs had the opposite consequence. An RS contrast of the neoplasm histologic grade showed that the RS in stages IIB-III-IV was significantly greater than that in stages I-II-IIA (Figure 4(c)). This part of the result suggests that a high-risk score has an adverse effect on prognosis, which may be caused by a decrease in immune score.

### 3.5. Nomogram Model Construction and Visualization

**Figure 5:**
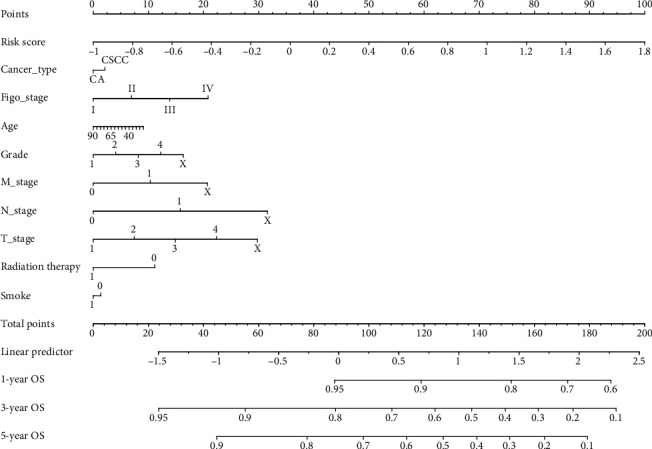
A nomogram based on the signature and clinical information.

The univariate and multivariate Cox regression analyses of clinicopathological characteristics and the four-IRL signature for the total TCGA dataset demonstrated that the four-IRL signature was an independent risk factor for CC patients (*p* < 0.05, Table 4). In univariate Cox analysis, FIGO stage and TNM stages were risk factors for CC patients (*p* < 0.05), whereas their prognostic values were not validated in the multivariate Cox analysis. Nonetheless, radiation therapy and a history of tobacco smoking were not correlated with prognosis independently. To better predict prognosis at one-, three-, and five-year OS of CC patients, we constructed a nomogram of variables such as the four-IRL signature, age, and FIGO stage (Figure 5).

### 3.6. Gene Set Enrichment Analysis

**Figure 6:**
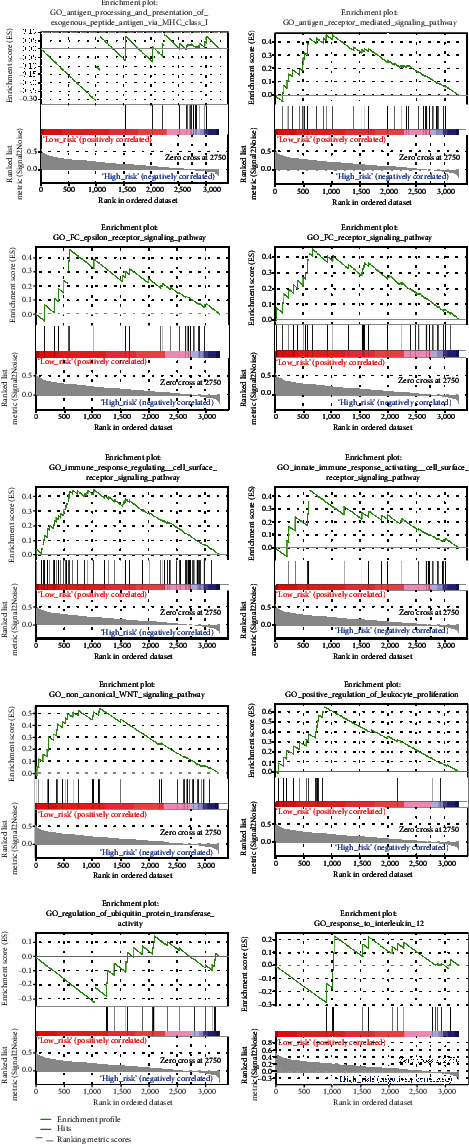
Immunologic characteristics regulated via the GSEA.

GSEA analysis of the two risk groups was carried out to predict enrichment status disparities of molecular mechanism functions. The enrichment analysis showed that 118 biological functions were markedly enriched in the low-risk group, whereas only one biological function (GO_ATP_SYNTHESIS_COUPLED_ELECTRON_TRANSPORT) was enriched in the high-risk group. In the enrichment status enriched in the low-risk group, we sought out couple immune-related responses as shown in Figure 6.

### 3.7. Evaluation of Infiltrating Immune Cells

**Figure 7:**
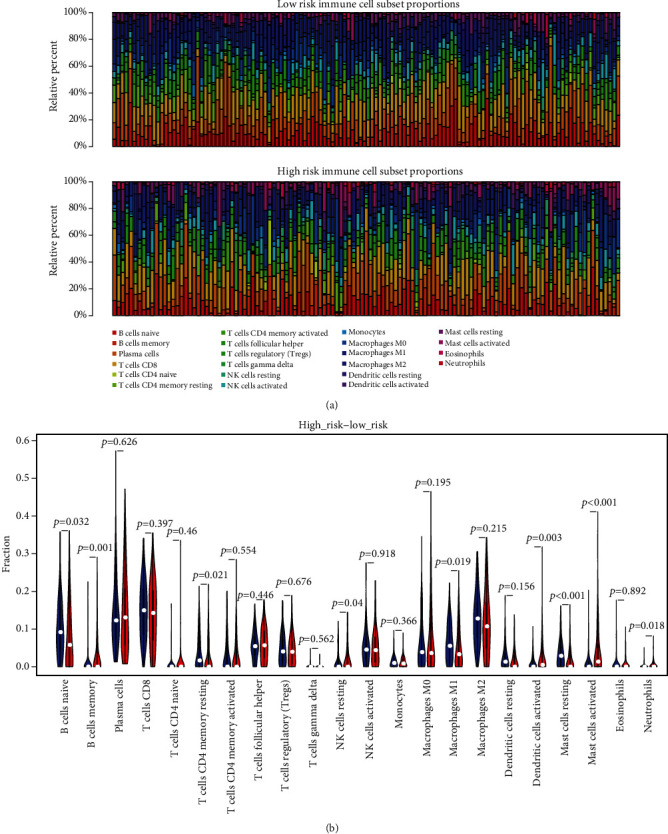
Evaluation of tumor-infiltrating immune cells. (a) The landscape of immune infiltration in risk groups. (b) The difference of 22 tumor-infiltrating immune cells among risk groups.

Previous studies have shown that infiltrating immune cells are closely related to the prognosis and treatment of malignant tumors [29]. From the GSEA analysis in our study, we discovered that the four-IRL signature was associated with many immune characteristics. Hence, the abundance of twenty-two infiltrating immune cells (Figure 7(a)) were estimated which showed that the abundance of nine infiltrating immune cells (B cell naïve, B cell memory, T cell CD4 memory resting, NK cells resting, macrophages M1, dendritic cells activated, mast cells resting, mast cells activated, and neutrophils) was significantly different (*p* < 0.05) between the risk groups (Figure 7(b)). The abundance of four infiltrating immune cells (B cell naïve, T cell CD4 memory resting, macrophages M1, and mast cells resting) in the low-risk group were significantly higher than that in the high-risk group as shown by Student's *t*-test (*p* < 0.05).

### 3.8. Construction of an IRL-Associated ceRNA Network

**Figure 8:**
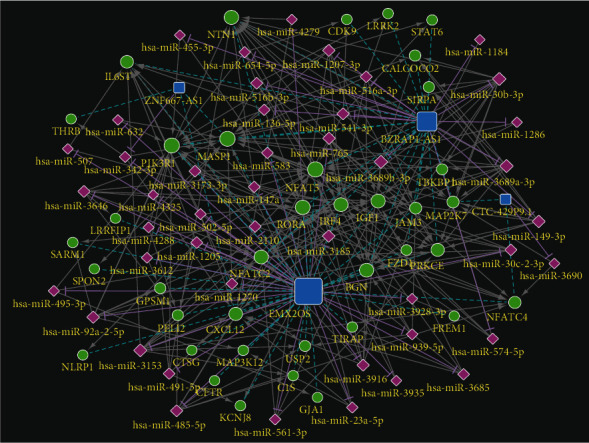
ceRNA network of 4-IRLs.

**Figure 9:**
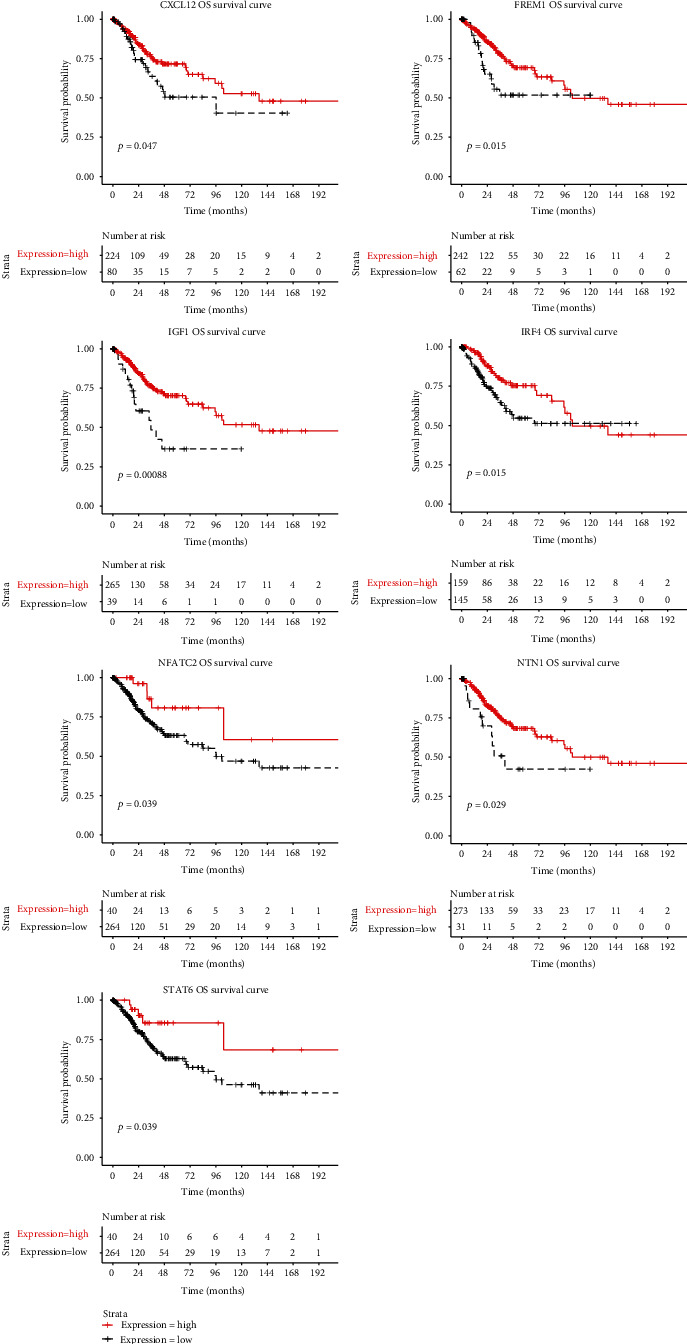
Kaplan–Meier curve of 7 mRNAs.

Four-IRLs, forty-five hub mRNAs, and thirty-eight miRNAs were involved in the ceRNA network (Figure 8). A total of 46 lncRNA-miRNA pairs, 232 miRNA-mRNA pairs, and 55 lncRNA-mRNA pairs were identified. The downregulation of 12 mRNAs in the ceRNA network was significantly related to OS in CC. Among them, the downregulation of seven mRNAs (CXCL12, FREM1, IGF1, IRF4, NFATC2, NTN1, and STAT6) had an adverse effect on prognosis, which was in line with what was expected (Figure 9).

## 4. Discussion

In this study, a cohort of 326 CC and 21 normal tissue samples from two datasets (TCGA, GSE6791) were included to identify the differential lncRNAs for patients with CC. A total of 1040 IRGs were collected from the InnateDB. Four IRLs (BZRAP1-AS1, EMX2OS, ZNF667-AS1, and CTC-429P9.1) were identified after ssGSEA analysis, Pearson correlation coefficient analysis, univariate Cox regression analysis, and Kaplan–Meier survival analysis between the lncRNAs and IS. The prognostic risk model based on the four IRLs could divide CC patients into two risk groups according to the median RS, which was validated by dividing the total samples equally. The univariate and multivariate cox regression analyses of clinicopathological characteristics showed that age, AJCC stage, and the four-IRL signature were all independent prognostic factors. A nomogram was constructed based on age, AJCC stage, and four-IRL signature to predict OS for CC patients more clearly. We also found that RS and IS showed significant negative correlation, which indicated that high RS had an adverse effect on prognosis due to a decrease in IS.

In our study, the four IRLs (BZRAP1-AS1, EMX2OS, ZNF667-AS1, and CTC-429P9.1) were identified to be protective against CC; only ZNF667-AS1 had been previously reported in CC. In the existing literature, ZNF667-AS1 with low expression has been identified to be negatively correlated with the OS, tumor size, and FIGO stage in CC [30]. Additionally, ZNF667-AS1 can competitively bind to miR-93-3p, which targets PEG3, to regulate the progression of CC [31]. Recent research has shown that inhibiting PEG3 would promote the immune escape of cancer cells [32]. BZRAP1-AS1 was found to be a novel biomarker associated with prostate cancer (PC), being downregulated in PC samples [33]. It was shown, however, to be highly expressed in hepatocellular carcinoma and inhibited the transcription of THBS1 by recruiting DNMT3b to its promoter region [34]. Previous research has revealed that the downregulation of EMX2OS in classical papillary thyroid cancer might independently predict shorter recurrence-free survival [35], while the overexpression of EMX2OS in ovarian cancer and EMX2OS/miR-654/AKT3 axis may target PD-L1 (programmed cell death protein 1) to suppress the initiation and progression of cancer [36]. Accumulating evidences have suggested that Thrombospondin-1 (THBS1) may affect tumor immunity [37]. PD1-PDL1 (PD1 ligand) has already been shown to be an important immune checkpoint pathway, which can be used by cancer cells to evade immune attacks [38]. Thus, BZRAP1-AS1 and EMX2OS may play a dual role in cancer and directly or indirectly regulate tumor immunology. By contrast, no reports concerning CTC-429P9.1 in cancer have been published, and therefore, the role of CTC-429P9.1 remains unclear.

In recent years, a small number of researches have reported that lncRNAs can directly or indirectly affect the tumor microenvironment of cervical cancer. LOC105374902 induced by TNF-*α*, a multiple functional cytokine which can regulate inflammation and immunity of cancer, was found to promote the malignant behavior of cervical cancer cells by acting as a sponge of miR-1285-3p [39]. lncRNA SNHG14 was shown to be associated with the activation of the JAK-STAT pathway in cervical tumor cells [40]. STAT3-binding sequence in the enhancer region of lncRNA MALAT1 was demonstrated to be crucial for the IL-6- or STAT3-induced MALAT1 promoter activation in cervical cancer HeLa cells [41]. HOTAIR was identified to promote the overactivation of the Wnt/*β*-catenin signaling pathway by the downregulation of PCDH10, SOX17, AJAP1, and MAGI2 and also TET [42]. lncRNA HIPK1-AS has been proved to regulate the inflammatory process of cervical cancer [43]. Generally, there are still relatively few immune-related lncRNAs in CC.

Our GSEA analysis showed that certain immune-related enrichment statuses were dramatically enriched in the low-risk group. Noncanonical WNT signaling has been demonstrated to be closely associated with cancer stem cell survival, bulk tumor expansion, and invasion/metastasis, which have the potential for tumor immunology [44]. Ubiquitination has been shown to be crucial for tumor immunity [45].

The ceRNA network was established to explore specific mechanisms in the development of CC based on the competing endogenous RNA theory. Combining the mRNAs of the most outstanding prognosis with the connectivity of miRNAs, the following ceRNA relationships have been established, which should be used as a follow-up verification: EMX2OS/hsa-miR-3153/CXCL12, EMX2OS/hsa-miR-3928-3p/FREM1, BZRAP1-AS1/hsa-miR-30b-3p/IGF1, EMX2OS/hsa-miR-92a-2-5p/IRF4, BZRAP1-AS1/hsa-miR-30b-3p/NFATC2, BZRAP1-AS1/hsa-miR-30b-3p/NTN1, and BZRAP1-AS1/hsa-miR-541-3p/STAT6. CXCL12 binds primarily to CXCR4 which can cause a mass of signal routing including the immunity [46]. FREM1 has been authenticated as an immune-related gene which may be a potential target for immunotherapy in breast cancer and clear cell renal cell carcinoma [47, 48]. The insulin-like growth factor 1 (IGF1) pathway has been proven to contribute to the suppression of immune tumor microenvironment (TME) in gynecologic cancers [49]. IRF4^+^ Tregs have been confirmed to be correlated with poor prognosis in patients with multiple cancers [50]. NFATC2 regulates IL-21 expression in human CD4+ CD45RO+ T lymphocytes [51]. STAT6 is a factor converge on intracellular determinants of cell functions and drives the recruitment and polarization of tumor-associated macrophages (TAMs) [52]. Thus, our IRLs need to be further investigated.

Compared to a certain number of studies carried out to explore IRL signatures in several human malignancies [53–59], this was the first study based on a comprehensive analysis that focused on IRL signature in CC. However, our study has some limitations. Additional datasets are needed to validate our study. The specific molecular mechanisms in which these genes may be involved should be verified through in vitro and in vivo experiments.

## 5. Conclusions

We established a four-IRL-based signature with a prognostic value for CC, which could stratify CC patients into the low- and high-risk groups. Meanwhile, the specific mechanisms of the four IRLs in the development of CC were preliminarily ascertained. This may provide the basis for tumor prevention and immunotherapy in the future.

## Figures and Tables

**Table 1:** Differential mRNAs and lncRNAs.

	mRNA	lncRNA
TCGA		
Up	3305	472
Down	4163	733
Total	7468	1205
GSE6791		
Up	4680	95
Down	3133	695
Total	7813	790

**Table 2:** K-M analysis, univariate Cox regression analysis, and Pearson correlation analysis of 4-IRLs.

lncRNA	K-M analysis	Univariate Cox regression analysis				Pearson correlation analysis		Differential expression analysis
	*p* value	HR	Lower.95	Upper.95	*p* value	*r*	*p*.adj.value	Up_down
BZRAP1-AS1	0.0002	0.82	0.72	0.93	0.00	0.32	2.84*E*-08	Down_lnc
EMX2OS	0.0010	0.91	0.82	1.00	0.05	-0.25	3.01*E*-05	Down_lnc
ZNF667-AS1	0.0019	0.87	0.77	0.99	0.03	-0.17	0.007	Down_lnc
CTC-429P9.1	0.0159	0.93	0.82	1.06	0.27	-0.24	5.15*E*-05	Down_lnc

**Table 3:** *β* of each of the 4 IRLs.

Ensemble_ID	lncRNA	*β*
ENSG00000166770.10	ZNF667-AS1	-0.152565
ENSG00000229847.8	EMX2OS	-0.019887
ENSG00000265148.5	BZRAP1-AS1	-0.17831
ENSG00000269427.1	CTC-429P9.1	0.0096755

**Table 4:** Univariate and multivariate Cox regression analyses of clinical parameters.

Variables	Univariate			Multivariate		
	Coeff	HR (95% CI)	*p* value	Coeff	HR (95% CI)	*p* value
Risk score	0.854	2.349 (1.317-4.191)	0.004	0.819	2.268 (1.101-4.674)	0.026
Age	0.014	1.014 (0.996-1.032)	0.117	-0.003	0.997 (0.973-1.022)	0.812
Cancer type	0.040	1.041 (0.531-2.041)	0.907	0.049	1.050 (0.452-2.438)	0.910
FIGO stage	0.362	1.436 (1.141-1.806)	0.002	0.159	1.173 (0.834-1.648)	0.359
Tumor grade	0.216	1.241 (0.969-1.589)	0.087	0.093	1.098 (0.82-1.47)	0.530
M stage	0.419	1.520 (1.130-2.044)	0.006	0.237	1.268 (0.89-1.806)	0.188
N stage	0.674	1.962 (1.406-2.738)	<0.001	0.362	1.436 (0.881-2.341)	0.146
T stage	0.398	1.488 (1.222-1.813)	<0.001	0.171	1.186 (0.872-1.613)	0.277
Radiotherapy	0.289	1.335 (0.735-2.426)	0.343	-0.256	0.774 (0.368-1.629)	0.500
Smoke	0.130	1.139 (0.696-1.862)	0.605	-0.031	0.970 (0.484-1.943)	0.930

## Data Availability

The RNA sequencing profiles are able to be gained from The Cancer Genome Atlas (TCGA) (https://toil.xenahubs.net) and Gene Expression Omnibus (GEO) (http://www.ncbi.nlm.nih.gov/geo/). The immune-related genes can be downloaded from the InnateDB database (http://www.innatedb.com).

## Abbreviations

CC: Cervical cancer
IRL: Immune-related lncRNA
IS: Immune score
ssGSEA: Single-sample gene set enrichment analysis
OS: Overall survival
AUC: Area under the curve
ROC: Receiver operating characteristic
HPV: Human papillomavirus
EMT: Epithelial-mesenchymal transition
GSEA: Gene set enrichment analysis
TCGA: The Cancer Genome Atlas
GEO: Gene expression omnibus
HR: Hazard ratio.
